# Establishing ADC-Based Histogram and Texture Features for Early Treatment-Induced Changes in Head and Neck Squamous Cell Carcinoma

**DOI:** 10.3389/fonc.2021.708398

**Published:** 2021-09-02

**Authors:** Anna Rodrigues, Kelly Loman, Jeff Nawrocki, Jenny K. Hoang, Zheng Chang, Yvonne M. Mowery, Taofik Oyekunle, Donna Niedzwiecki, David M. Brizel, Oana Craciunescu

**Affiliations:** ^1^Department of Radiation Oncology, Duke University Medical Center, Durham, NC, United States; ^2^Department of Radiology, Johns Hopkins University, Baltimore, MD, United States; ^3^Department of Head and Neck Surgery and Communication Sciences, Duke University Medical Center, Durham, NC, United States

**Keywords:** texture analysis, DW-MRI, early treatment assessment, head and neck cancer, repeatability

## Abstract

The purpose of this study was to assess baseline variability in histogram and texture features derived from apparent diffusion coefficient (ADC) maps from diffusion-weighted MRI (DW-MRI) examinations and to identify early treatment-induced changes to these features in patients with head and neck squamous cell carcinoma (HNSCC) undergoing definitive chemoradiation. Patients with American Joint Committee on Cancer Stage III–IV (7^th^ edition) HNSCC were prospectively enrolled on an IRB-approved study to undergo two pre-treatment baseline DW-MRI examinations, performed 1 week apart, and a third early intra-treatment DW-MRI examination during the second week of chemoradiation. Forty texture and six histogram features were derived from ADC maps. Repeatability of the features from the baseline ADC maps was assessed with the intra-class correlation coefficient (ICC). A Wilcoxon signed-rank test compared average baseline and early treatment feature changes. Data from nine patients were used for this study. Comparison of the two baseline ADC maps yielded 11 features with an ICC ≥ 0.80, indicating that these features had excellent repeatability: Run Gray-Level Non-Uniformity, Coarseness, Long Zone High Gray-Level, Variance (Histogram Feature), Cluster Shade, Long Zone, Variance (Texture Feature), Run Length Non-Uniformity, Correlation, Cluster Tendency, and ADC Median. The Wilcoxon signed-rank test resulted in four features with significantly different early treatment-induced changes compared to the baseline values: Run Gray-Level Non-Uniformity (p = 0.005), Run Length Non-Uniformity (p = 0.005), Coarseness (p = 0.006), and Variance (Histogram) (p = 0.006). The feasibility of histogram and texture analysis as a potential biomarker is dependent on the baseline variability of each metric, which disqualifies many features.

## Introduction

Recent publications have summarized the current state of radiomics research utilizing functional imaging in head and neck cancer for tumor segmentation, prognostic and predictive response biomarkers, and monitoring of normal tissue sequelae (1, 2). While ^18^F-fluorodeoxyglucose positron emission tomography (^18^FDG-PET) can provide information on tumor metabolic activity before, during, and after definitive radiation or chemoradiation (CRT), its sensitivity to inflammation and poor spatial resolution pose significant limitations for imaging during therapy (3).

Diffusion-weighted magnetic resonance imaging (DW-MRI) has garnered much interest in the past decade as an imaging biomarker for cancer treatment response with the potential to detect early treatment-induced changes (4–9). DW-MRI-derived apparent diffusion coefficient (ADC) maps express water molecular motion, which tends to be relatively low in tumors due to their higher cellular density than normal tissue. CRT has been shown to increase water molecular motion in tumors undergoing a favorable treatment response (10). Most DW-MRI studies have investigated first-order histogram features, such as ADC change of tumors between pre-treatment and post-treatment examinations or pre-, intra-, and post-treatment examinations (7, 11–13). Vandecaveye et al. determined that percentage change in ADC from 3 weeks post-CRT relative to baseline examination was significantly correlated with patient outcome, allowing for early assessment of treatment response (12). In a similar study by Kim et al. that also included early intra-treatment examinations (1 week after treatment began), the change in ADC between baseline and intra-treatment examinations had the highest sensitivity for differentiating complete and partial responders (11). These studies have shown the viability of using DW-MRI in the setting of detecting response during or shortly after completion of treatment by utilizing first-order histogram imaging features. Beyond the use of first-order ADC histogram features such as mean ADC, recent studies have evaluated the use of second-order texture features for response prediction (1, 2, 14, 15).

An important aspect of interpreting these early treatment-induced changes is understanding the underlying baseline repeatability of the features derived from the ADC maps (5). The National Cancer Institute-sponsored consensus conference on DW-MRI identified baseline repeatability as an important parameter needing evaluation (5). To date, our group has been the only one to examine baseline mean ADC variability for HNSCC (16). The repeatability coefficient for baseline nodal ADC was 15%, and the authors emphasized the importance of accounting for inherent baseline variability when assessing treatment-induced changes during CRT. These studies suggest that texture features can be used to quantify treatment-induced changes, which may correlate with histologic changes in MR studies (7).

The primary purpose of this study was to establish baseline repeatability of histogram and texture features from baseline pre-treatment DW-MRI-derived ADC maps. A secondary goal was identification of a subset of histogram and texture features that significantly changed between baseline and early treatment DW-MRI-derived ADC maps. We hypothesized that features with a high baseline repeatability would be candidates for further evaluation as features that could predict early treatment-induced changes.

## Materials and Methods

### Patient Cohort and Clinical Protocol

Patients in this IRB-approved retrospective study were adults undergoing concurrent CRT for head and neck squamous cell carcinoma (AJCC stages II–IVB, 7^th^ edition). Patients received intensity-modulated radiation therapy (IMRT) to a total dose of 70 Gy at 2 Gy/fraction (6MV photons, between 5 and 11 fields) with concurrent cisplatin and targeted therapy with bevacizumab and erlotinib and represented a subset of the overall patient population. The outcome of this trial has been reported elsewhere (clinicaltrials.gov: NCT00140556) (17). Written informed consent was obtained from all patients.

The purpose of this study was to investigate the application of DW-MRI for tumor physiologic assessment of patients undergoing concurrent chemoradiation for locally advanced head and neck cancer (16–18). The goal of this study was to evaluate imaging texture features for prognostic potential. Each patient underwent two baseline (baseline 1 and baseline 2) DW-MRI examinations, 1 week apart, to establish baseline intrinsic variability and a third examination (early treatment) performed in the second week of CRT. DW-MRI data generated ADC maps for the nodal disease were analyzed.

### MR Imaging Protocol

MRI data were acquired on a 1.5-T system (Signa EXCITE, GE Healthcare [software version 14x and 15x], Fairfield, CT) with a bird-cage quadrature head coil with a head and neck immobilization designed during the CT simulation.

DW-MRI examinations were acquired axially using a spin-echo planar imaging (EPI) sequence: TR = 10,000 ms, TE = 64 ms, field of view (FOV) = 240 × 240 mm^2^, matrix size = 256 × 256, slice thickness = 5 mm, gap = 0 mm, and 2 averages. Examinations were acquired using diffusion b-values of 0, 250, 500, and 1000 s/mm^2^ applied in six directions. ADC maps for each DW-MRI examination were generated offline using an in-house-developed research software program.

### Image Processing and ROI Selection

For the retrospective analysis, ADC maps were rigidly registered to their temporally corresponding CT examination (baseline 1, baseline 2, or early intra-treatment) using an image registration software (Velocity AI, Velocity Medical Solutions). Nodal volumes were contoured by experienced radiation oncologists (DB, DY, YM). Following registration of the ADC map to CT, the functional image was resampled to the CT. After resampling, the ADC maps were updated to the resampled CT resolution.

### Histogram and Texture Features

Six histogram and 40 texture features were calculated on all resampled ADC maps for the nodal ROIs. These features are summarized in **Table 1**.

**Table 1:** List of 46 features (40 texture features and 6 histogram features) investigated for this study.

Features	
Gray-Level Co-occurrence Matrix (GLCM) (N = 13)	Energy
	Entropy
	Correlation
	Contrast
	Homogeneity (Haralick’s)
	Variance
	Sum Mean
	Inertia
	Cluster Shade
	Cluster Tendency
	Maximum Probability
	Inverse Variance
	Homogeneity (Tixier)
Gray-level Run Length Matrix (GLRLM) (N = 11)	Short Run
	Long Run
	Low Gray-Level Run
	High Gray-Level Run
	Short Run Low Gray-Level
	Short Run High Gray-Level
	Long Run Low Gray-Level
	Long Run High Gray-Level
	Run Gray-Level Non-Uniformity
	Run Length Non-Uniformity
	Run Percentage
Gray-level Size Zone Matrix (GLSZM) (N = 11)	Short Zone
	Long Zone
	Low Gray-Level Zone
	High Gray-Level Zone
	Short Zone Low Gray-Level
	Short Zone High Gray-Level
	Long Zone Low Gray-Level
	Long Zone High Gray-Level
	Zone Gray-Level Non-Uniformity
	Zone Size Non-Uniformity
	Zone Percentage
Neighborhood Gray-level Difference Matrix (NGLDM) (N = 5)	Coarseness
	Contrast
	Busyness
	Complexity
	Texture Strength
Histogram features (N = 6)	ADC Max
	ADC Mean
	ADC Median
	Skewness
	Kurtosis
	Variance

Texture analysis was accomplished using in-house software (19) to calculate four mathematical matrices representing the spatial distribution of voxels in an image: Gray-Level Co-occurrence Matrix (GLCM), Gray-Level Run Length Matrix (GLRLM), Gray-Level Size Zone Matrix (GLSZM), and the Neighborhood Gray-Level Difference Matrix (NGLDM) (20–24). Texture analysis requires discrete values in order to tabulate the gray levels into matrices. Further, the voxels must be resampled to a normalized value between patient image sets. Resampling to 64 (bit depth of 6-bit) was used in this study. Thirteen angles were used to calculate the texture features. GLCM features are used to quantify coarseness *vs*. smoothness within an image by calculating how often pairs of voxels having specific values with a specific spatial relationship occur within an image. GLRLM is similar to GLCM, but instead looks at lengths of voxels rather than pairs. Therefore, GLRLM, while still considering coarseness, focuses on the lengths of similar high- and low-intensity gray levels within an image. GLSZM identifies zones of uniform voxels within an image, as opposed to lengths. GLSZM calculates the occurrences of a specific gray level found in zones, or groups, regardless of size. GLSZM is useful for identifying the homogeneity of an image. NGLDM features are used to investigate the variation found between texture zones in an image.

Texture features representing characteristics of the image are derived from these texture matrices: 13 local features from the GLCM, 11 regional features from the GLRLM, 11 regional features from the GLSZM, and 5 local features from the NGLDM.

### Statistical Methods

To identify features with high repeatability between baseline studies (baseline 1 and baseline 2), we compared all histogram and texture features using the intra-class correlation coefficient (ICC). Variance between baselines reflects inherent imprecision of the instrument used for measuring as well as any intrinsic baseline variation. An ICC cutoff value of at least 0.8 was chosen, indicating that the metric had excellent repeatability (25).

For features with highest repeatability with an ICC ≥ 0.80, the Wilcoxon signed-rank test was used to test if the difference between the mean of the baselines and the early treatment for a given metric was nonzero, indicating a treatment-induced change. A Holm–Bonferroni correction was applied to control for the familywise error rate (26). An adjusted p-value was thus calculated for each metric. All statistical analyses were performed using SAS 9.4 (SAS Institute, Inc. Cary, NC).

## Results

### Patient Cohort and Clinical Protocol

Nine patients were evaluated in this study, corresponding to patient numbers 1, 2, 3, 4, 5, 7, 8, 12, and 14 in Table 1 by Hoang et al. (16) Seven patients were excluded from this analysis: three with missing/incomplete CT data (patient numbers 6, 9, and 11), three with image registration problems (patient numbers 10, 13, and 16), and one excluded during statistical analysis as an outlier (patient number 15). For these nine patients, all except one (number 7) were complete responders to treatment and did not recur subsequently. **Table 2** lists the nodal volumes size for the patients evaluated in this study.

**Table 2:** Nodal volumes for the evaluated patients..

Patient	ADC baseline 1	ADC baseline 2	ADC early treatment
	Nodal volume (cm^3^)	Nodal volume (cm^3^)	Nodal volume (cm^3^)
1	21.35	18.30	8.04
2	8.36	10.53	0.98
3	6.61	5.53	5.11
4	5.81	7.09	2.83
5	14.72	19.93	3.50
7	7.40	8.78	7.37
8	9.05	10.71	5.99
12	4.57	4.25	2.98
14	5.34	6.69	4.49

The patient number corresponds to the study by Hoang et al. in **Table 1**

### Baseline Histogram and Texture Feature Repeatability

The results of the ICC for baseline histogram and texture features are shown in **Table 3**. Eleven features showed an ICC ≥ 0.8, indicating that these features had excellent repeatability:

- Four features from the Gray-Level Co-occurrence Matric (GLCM): Correlation (0.83), Variance (0.85), Cluster Shade (0.88), Cluster Tendency (0.81);
- Two features from the Gray-Level Run Length Matrix (GLRLM): Run Gray-Level Non-Uniformity (0.95) and Run Length Non-Uniformity (0.85);
- Two features from the Gray-Level Size Zone Matrix (GLSZM): Long Zone (0.86) and Long Zone High Gray-Level (0.94);
- One feature from the Neighborhood Gray-Level Difference Matrix (NGLDM): Coarseness (0.94);
- Two features from the Histogram: ADC Median (0.8), and Variance (0.93).

**Table 3:** Intra-class correlation coefficient (ICC) for texture features from baseline 1 and 2. ICC ≥ 0.8 are bolded.

	Features	ICC
Gray-Level Co-occurrence Matrix (GLCM) (N = 13)	Energy	0.41
	Entropy	0.56
	**Correlation**	**0.83**
	Contrast	0.59
	Homogeneity (Haralick’s)	0.46
	**Variance**	**0.85**
	Sum Mean	0.39
	Inertia	0.59
	**Cluster Shade**	**0.88**
	**Cluster Tendency**	**0.81**
	Max Probability	0.25
	Inverse Variance	0.53
	Homogeneity (Tixier)	0.48
Gray-level Run Length Matrix (GLRLM) (N = 11)	Short Run	0.36
	Long Run	0.35
	Low Gray-Level Run	0.22
	High Gray-Level Run	0.24
	Short Run Low Gray-Level	0.24
	Short Run High Gray-Level	0.24
	Long Run Low Gray-Level	0.16
	Long Run High Gray-Level	0.31
	**Run Gray-Level N-Uniformity**	**0.95**
	**Run Length Non-Uniformity**	**0.85**
	Run Percentage	0.53
Gray-level Size Zone Matrix (GLSZM) (N = 11)	Short Zone	0.50
	**Long Zone**	**0.86**
	Low Gray-Level Zone	0.15
	High Gray-Level Zone	0.15
	Short Zone Low Gray-Level	0.10
	Short Zone High Gray-Level	0.11
	Long Zone Low Gray-Level	0.12
	**Long Zone High Gray-Level**	**0.94**
	Zone Gray-Level N-Uniformity	0.74
	Zone Length Non-Uniformity	0.44
	Zone Percentage	0.29
Neighborhood Gray-level Difference Matrix (NGLDM) (N = 5)	**Coarseness**	**0.94**
	Contrast	0.30
	Busyness	NE
	Complexity	NE
	Texture Strength	0.79
Histogram features (N = 6)	ADC Max	0.46
	ADC Mean	0.79
	**ADC Median**	**0.80**
	Skewness	0.47
	Kurtosis	0.40
	**Variance**	**0.93**

NE, not estimable.

### Baseline to Early Treatment Histogram and Texture Feature Significance

The results of the Wilcoxon signed-rank test for the paired data comparing average baseline to early treatment features are shown in **Table 4**. After application of the Holm–Bonferroni method to adjust the p-values, four features remained statistically significant: Run Gray-Level Non-Uniformity (p = 0.005), Run Length Non-Uniformity (p = 0.005), Coarseness (p = 0.006), and Variance (Histogram) (p = 0.006).

**Table 4:** Results of the Holm–Bonferroni-corrected Wilcoxon signed-rank test between the average baselines 1 and 2 and early treatment histogram and texture features.

Features	ICC	*p(_k_)*	$\frac{\alpha }{m+1-k}$	Median (Q1, Q3)	
		Wilcoxon signed-rank test	Holm–Bonferroni adjustment	Average (Baseline 1, Baseline 2)	Early treatment
Run Gray-Level Non-Uniformity	0.95	0.004	0.005	286.72 (188.59, 360.76)	98.46 (80.89, 200.40)
Run Length Non-Uniformity	0.85	0.004	0.005	4671.12 (4068.17, 6884.73)	2547.96 (2001.87, 3923.20)
Coarseness	0.94	0.004	0.006	75.07 (61.22, 103.28)	167.50 (98.24, 239.43)
Variance (Histogram)	0.93	0.004	0.006	25180.96 (10899.19, 38270.55)	2854.79 (1923.43, 11871.17)
ADC Median	0.80	0.008	0.007	4822.50 (4195.50, 5053.25)	5798.00 (5117.50, 6315.50)
Long Zone	0.86	0.10	0.008	343.09 (230.89, 766.42)	68.81 (26.15, 106.54)
Cluster Shade	0.88	0.20	0.010	2040.54 (1105.81, 5957.70)	1574.28 (808.08, 2212.27)
Correlation	0.83	0.25	0.013	0.013 (0.011,0.021)	0.014 (0.008, 0.017)
Long Zone High Gray-Level	0.94	0.43	0.017	76764.73 (38461.63, 20359.57)	54948.37 (15318.77, 92481.69)
Variance (GLCM)	0.85	0.65	0.025	56.60 (41.78, 66.15)	54.11 (47.24, 66.62)
Cluster Tendency	0.81	0.91	0.050	173957.12 (118161.33, 393835.65)	158679.37 (139103.70, 236846.15)

Bolded features are significant after Holm–Bonferroni adjustment.

**Figure 1:**
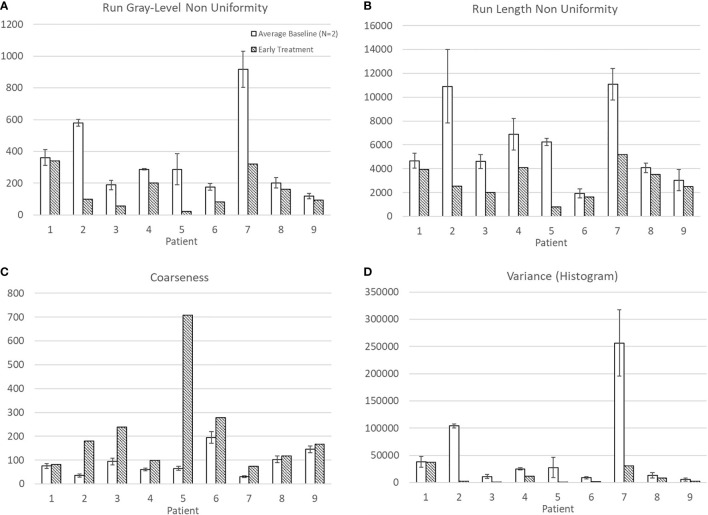
Average and standard deviation for baseline 1 & 2 and early treatment features with statistically significant differences: **(A)** Run Gray-Level Non Uniformity, **(B)** Run Length Non Uniformity, **(C)** Coarseness, and **(D)** Variance (Histogram).

Average baseline and early intra-treatment values for these four features for all nine patients are shown in **Figure 1**. For all nine patients, Run Gray-Level Non-Uniformity, Run Length Non-Uniformity, and Variance (Histogram) decreased between baseline and early treatment ADC maps. Coarseness increased for all nine patients between baseline and early intra-treatment ADC maps.

## Discussion

Notably, this is the first study to investigate baseline repeatability of histogram and texture features in ADC maps derived from baseline DW-MRI examinations in patients with head and neck squamous cell carcinoma. It also analyzed which of these features changed significantly early in the course of treatment relative to their baseline values. This study is unique as it established the variability of pre-treatment baseline DW-MRI ADC first-order histogram and second-order texture features. Of the 45 histogram and texture features, four features displayed excellent repeatability (ICC ≥ 0.8), and early treatment changes in their values were found to be significant: one first-order histogram feature (Variance) and three second-order texture features: two features from the GLRLM (Run Gray-Level Non-Uniformity, Run Length Non-Uniformity) and one feature from NGLDM (Coarseness).

**Figure 2:**
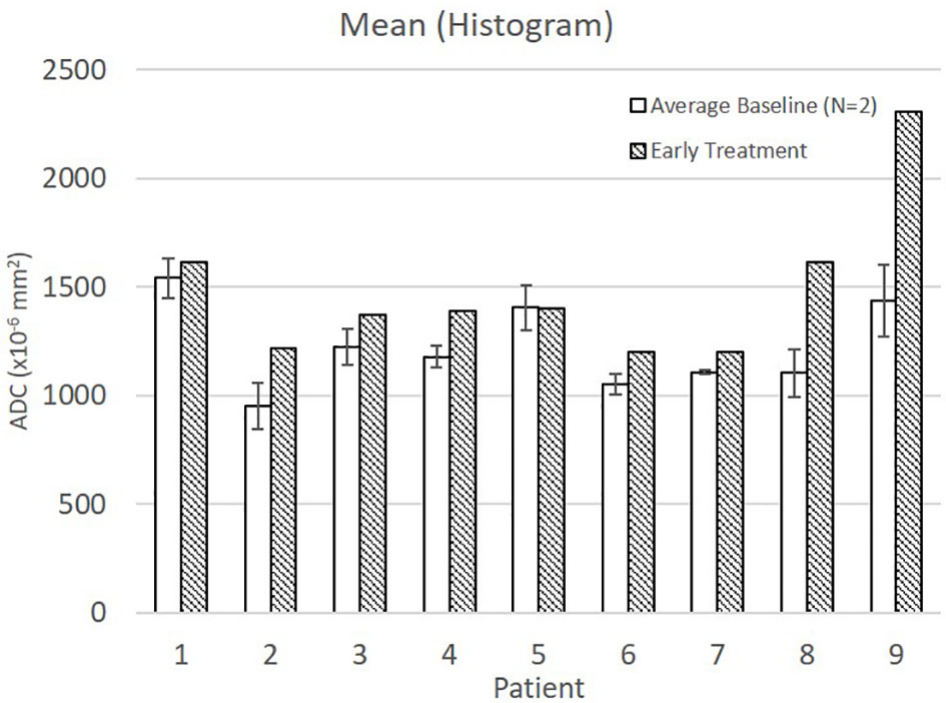
Average and standard deviation for baseline 1 & 2 and early treatment mean ADC.

Mean ADC increased from baseline to early treatment consistent with tumor death-associated decreased cellular density (**Figure 2**). Variance decreased between baseline and early treatment, indicating a narrower distribution of the ADC values, i.e., more voxels having similar values than they had at baseline (**Figure 1D**). Coupled with the increase in mean ADC values, the ADC values were more homogenous in the early intra-treatment examination.

Run Gray-Level Non-Uniformity measures the variability of gray-level intensity values in the ADC map. A lower Run Gray-Level Non-Uniformity thus indicates that neighboring voxels tend to have similar ADC intensity values, i.e., indicating a more homogenous diffusion coefficient of water outside the cell walls. For all patients in this study, the Run Gray-Level Non-Uniformity decreased between the baseline and early intra-treatment examinations (**Figure 1A**). Run Length Non-Uniformity measures the similarity of length runs where a lower value indicates more homogeneity among the run lengths in the image, i.e., more runs of voxels of the same length. For the nine patients, the Run Length Non-Uniformity decrease from baseline to early intra-treatment indicates more runs of voxels of the same length (**Figure 1B**). Coarseness is a measure of the average difference between the center voxel and its neighbor voxels and is an indication of the spatial frequency. A higher value indicates a lower spatial frequency and thus a locally more uniform texture. For the nine patients, the coarseness increased, i.e., the ADC maps displayed more uniform values across the ROIs (**Figure 1C**).

Interpreting the biological meaning of higher-order features such as texture features is not straightforward because these features usually cannot be explained by physiological models. However, the summarized texture feature results indicate that characteristics of the ADC map changed to make the values more homogeneous.

Limitations of this study include that it was a small, single-institution pilot study. Future research should include larger, multi-institutional data sets. Further, since the ADC measurements were obtained by a single radiologist, interobserver and intra-observer variability could not be assessed. Notably, texture features typically are not robust to dynamic range and matrix size; consistency in applied methodology in both imaging technique and analysis is key, especially when validating results across institutions (27, 28). Image acquisition parameters that can affect the robustness of the resulting texture feature include selection of b-value, repetition time, echo time, inhomogeneity, and receiver bandwidth (29). Multiple studies have shown that the range and number of b-values used to acquire DW-MRI can affect the resulting ADC maps. It is known that certain b-values can cause image distortions due to the chemical shift artifact (30). However, to date there is no study that has assessed the effect of b-value choice on the resulting texture features. b-value selection for this study was per our clinical acquisition protocol. The use of the same acquisition protocol for both baseline and early-treatment DW-MRI acquisitions on the same scanner negated the potential effect of varying b-values on the resulting texture features. Thus, clinical implementation requires that all DW-MRI scans be performed on the same scanner with the same protocol to remove any scanner-dependent variability in the resulting histogram and texture features (31). Other contributions to uncertainty in the baseline 1 and baseline 2 images can arise from patient-specific, physiological, and anatomical variations. A study on healthy subjects by Kolff-Gart et al. indicated that between-visit ADC values acquired 1 month apart varied the least if the subject is scanned on the same scanner with the same parameters (32).

Future work will include a larger patient database to investigate the value of changes in Run Gray-Level Non-Uniformity, Run Length Non-Uniformity, Coarseness, and Variance (Histogram) between baseline and early intra-treatment ADC maps in predicting early treatment response and nonresponse to CRT for patients with HNSCC.

In conclusion, lack of baseline repeatability disqualifies many histogram and texture features from DW-MRI-derived ADC maps for quantification of early treatment-induced changes in HNSCC patients. Furthermore, a subset of these ADC histogram and texture features shows significant changes after 1 week of CRT and warrants further study to provide quantitative assessment of early treatment changes for HNSCC using DW-MRI.

## Data Availability Statement

The raw data supporting the conclusions of this article will be made available by the authors, without undue reservation.

## Ethics Statement

The studies involving human participants were reviewed and approved by the Duke University Health System Institutional Review Board. The patients/participants provided their written informed consent to participate in this study.

## Author Contributions

Study concept contributions by KL, JH, ZC, DB, and OC. Study design contributions by AR, KL, JN, DB, and OC. Literature search contributions by AR, KL, YM, DB, and OC. Experimental study contributions by AR, KL, YM, DB, and OC. Data acquisition contributions by AR and KL. Data analysis by AR and KL. Statistical analysis by AR, KL, JN, TO, and DN. Manuscript preparation by AR, KL, YM, DB, and OC. Manuscript editing and review by all authors. All authors contributed to the article and approved the submitted version.

## Conflict of Interest

The authors declare that the research was conducted in the absence of any commercial or financial relationships that could be construed as a potential conflict of interest.

## Publisher’s Note

All claims expressed in this article are solely those of the authors and do not necessarily represent those of their affiliated organizations, or those of the publisher, the editors and the reviewers. Any product that may be evaluated in this article, or claim that may be made by its manufacturer, is not guaranteed or endorsed by the publisher.

## References

[1] Wong AJ Kanwar A Mohamed AS Fuller CD. Radiomics in Head and Neck Cancer: From Exploration to Application. Trans Cancer Res (2016) 5(4):371–82. 10.21037/tcr.2016.07.18 PMC6322843 30627523

[2] Jethanandani A Lin TA Volpe S Elhalawani H Mohamed ASR Yang P. Exploring Applications of Radiomics in Magnetic Resonance Imaging of Head and Neck Cancer: A Systematic Review. Front Oncol (2018) 8:131. 10.3389/fonc.2018.00131 29868465 PMC5960677

[3] McCollum AD Burrell SC Haddad RI Norris CM Tishler RB Case MA. Positron Emission Tomography With 18F-Fluorodeoxyglucose to Predict Pathologic Response After Induction Chemotherapy and Definitive Chemoradiotherapy in Head and Neck Cancer. Head Neck (2004) 26(10):890–6. 10.1002/hed.20080 15390197

[4] Thoeny HC Ross BD. Predicting and Monitoring Cancer Treatment Response With DW-MRI. J Magn Reson Imaging (2010) 32(1):2–16. 10.1002/jmri.22167 20575076 PMC2918419

[5] Padhani AR Liu G Koh DM Chenevert TL Thoeny HC Takahara T. Diffusion-Weighted Magnetic Resonance Imaging as a Cancer Biomarker: Consensus and Recommendations. Neoplasia (2009) 11(2):102–25. 10.1593/neo.81328 PMC2631136 19186405

[6] Yankeelov TE Lepage M Chakravarthy A Broome EE Niermann KJ Kelley MC. Integration of Quantitative DCE-MRI and ADC Mapping to Monitor Treatment Response in Human Breast Cancer: Initial Results. Magn Reson Imaging (2007) 25(1):1–13. 10.1016/j.mri.2006.09.006 17222711 PMC2634832

[7] King AD Chow KK Yu KH Mo FKF Yeung DKW Yuan J. Head and Neck Squamous Cell Carcinoma: Diagnostic Performance of Diffusion-Weighted MR Imaging for the Prediction of Treatment Response. Radiology (2013) 266(2):531–8. 10.1148/radiol.12120167 23151830

[8] Hamstra DA Rehemtulla A Ross BD. Diffusion Magnetic Resonance Imaging: A Biomarker for Treatment Response in Oncology. J Clin Oncol (2007) 25(26):4104–9. 10.1200/JCO.2007.11.9610 17827460

[9] Brown AM Nagala S McLean MA Lu Y Scoffings D Apte A. Multi-Institutional Validation of a Novel Textural Analysis Tool for Preoperative Stratification of Suspected Thyroid Tumors on Diffusion-Weighted MRI. Magn Reson Med (2016) 75(4):1708–16. 10.1002/mrm.25743 PMC4654719 25995019

[10] Kauppinen RA. Monitoring Cytotoxic Tumour Treatment Response by Diffusion Magnetic Resonance Imaging and Proton Spectroscopy. NMR Biomed (2002) 15(1):6–17. 10.1002/nbm.742 11840548

[11] Kim S Loevner L Quon H Sherman E Weinstein G Kilger A. Diffusion-Weighted Magnetic Resonance Imaging for Predicting and Detecting Early Response to Chemoradiation Therapy of Squamous Cell Carcinomas of the Head and Neck. Clin Cancer Res (2009) 15(3):986–94. 10.1158/1078-0432.CCR-08-1287 PMC2673914 19188170

[12] Vandecaveye V Dirix P De Keyzer F Op De Beeck K Vander Poorten V Hauben E. Diffusion-Weighted Magnetic Resonance Imaging Early After Chemoradiotherapy to Monitor Treatment Response in Head-and-Neck Squamous Cell Carcinoma. Int J Radiat Oncol Biol Phys (2012) 82(3):1098–107. 10.1016/j.ijrobp.2011.02.044 21514067

[13] Vandecaveye V Dirix P De Keyzer F Op De Beeck K Vander Poorten V Roebben I. Predictive Value of Diffusion-Weighted Magnetic Resonance Imaging During Chemoradiotherapy for Head and Neck Squamous Cell Carcinoma. Eur Radiol (2010) 20(7):1703–14. 10.1007/s00330-010-1734-6 20179939

[14] Materka A. Texture Analysis Methodologies for Magnetic Resonance Imaging. Dialogues Clin Neurosci (2004) 6(2):243–50. 10.31887/DCNS.2004.6.2/amaterka PMC3181797 22033841

[15] Herlidou-Même S Constans JM Carsin B Olivie D Eliat PA Nadal-Desbarats L. MRI Texture Analysis on Texture Test Objects, Normal Brain and Intracranial Tumors. Magn Reson Imaging (2003) 21(9):989–93. 10.1016/S0730-725X(03)00212-1 14684201

[16] Hoang JK Choudhury KR Chang J Craciunescu OI Yoo DS Brizel DM. Diffusion-Weighted Imaging for Head and Neck Squamous Cell Carcinoma: Quantifying Repeatability to Understand Early Treatment-Induced Change. Am J Roentgenol (2014) 203(5):1104–8. 10.2214/AJR.14.12838 25341151

[17] Yoo DS Kirkpatrick JP Craciunescu O Broadwater G Peterson BL Carroll MD. Prospective Trial of Synchronous Bevacizumab, Erlotinib, and Concurrent Chemoradiation in Locally Advanced Head and Neck Cancer. Clin Cancer Res (2012) 18(5):1404–14. 10.1158/1078-0432.CCR-11-1982 22253412

[18] Onxley JD Yoo DS Muradyan N Macfall JR Brizel DM Craciunescu OI. Comprehensive Population-Averaged Arterial Input Function for Dynamic Contrast-Enhanced Vmagnetic Resonance Imaging of Head and Neck Cancer. Int J Radiat Oncol Biol Phys (2014) 89(3):658–65. 10.1016/j.ijrobp.2014.03.006 24929169

[19] Nawrocki J. Characterization of Gynecological Tumors Using Texture Analysis in the Context of an 18F-FDG Adaptive PET Protocol. Duke Univ (2015). 10.1118/1.4924335

[20] Galloway MM. Texture Analysis Using Gray Level Run Lengths. Comput Graph Image Process (1975) 4(2):172–9. 10.1016/S0146-664X(75)80008-6

[21] Haralick RM Dinstein I Shanmugam K. Textural Features for Image Classification. IEEE Trans Syst Man Cybern (1973) (6):610–21. 10.1109/TSMC.1973.4309314

[22] Amadasun M King R. Texural Features Corresponding to Texural Properties. IEEE Trans Syst Man Cybern (1989) 19(5):1264–74. 10.1109/21.44046

[23] Xu DH Kurani AS Furst JD Raicu DS. Run-Length Encoding for Volumetric Texture. Heart (2004) 27(25):452–8.

[24] Thibault G Fertil B Navarro C Pereira S Cau P Levy N. Texture Indexes and Gray Level Size Zone Matrix Application to Cell Nuclei Classification. 10th International Conference on Pattern Recognition and Information Processing (2009).

[25] Koo TK Li MY. A Guideline of Selecting and Reporting Intraclass Correlation Coefficients for Reliability Research. J Chiropr Med (2016) 15(2):155–63. 10.1016/j.jcm.2016.02.012 PMC4913118 27330520

[26] Holm S. A Simple Sequentially Rejective Multiple Test Procedure. Scand J Stat (1979) 65–70.

[27] Molina D Pérez-Beteta J Martínez-González A Martino J Velasquez C Arana E. Lack of Robustness of Textural Measures Obtained From 3D Brain Tumor MRIs Impose a Need for Standardization. PloS One (2017) 12(6):e0178843. 10.1371/journal.pone.0178843 28586353 PMC5460822

[28] Löfstedt T Brynolfsson P Asklund T Nyholm T Garpebring A. Gray-Level Invariant Haralick Texture Features. PloS One (2019) 14(2):e0212110. 10.1371/journal.pone.0212110 30794577 PMC6386443

[29] Brynolfsson P Nilsson D Torheim T Asklund T Karlsson CT Trygg J. Haralick Texture Features From Apparent Diffusion Coefficient (ADC) MRI Images Depend on Imaging and Pre-Processing Parameters. Sci Rep (2017) 7(1):4041. 10.1038/s41598-017-04151-4 PMC5481454 28642480

[30] Galbán CJ Mukherji SK Chenevert TL Meyer CR Hamstra DA Bland PH. A Feasibility Study of Parametric Response Map Analysis of Diffusion-Weighted Magnetic Resonance Imaging Scans of Head and Neck Cancer Patients for Providing Early Detection of Therapeutic Efficacy. Transl Oncol (2009) 2(3):184–90. 10.1593/tlo.09175 PMC2730136 19701503

[31] Huo J Alger J Kim H Brown M Okada K Pope W. Between-Scanner and Between-Visit Variation in Normal White Matter Apparent Diffusion Coefficient Values in the Setting of a Multi-Center Clinical Trial. Clin Neuroradiol (2016) 26(4):423–30. 10.1007/s00062-015-0381-3 25791203

[32] Kolff-Gart AS Pouwels PJW Noij DP Ljumanovic R Vandecaveye V De Keyzer F. Diffusion-Weighted Imaging of the Head and Neck in Healthy Subjects: Reproducibility of ADC Values in Different MRI Systems and Repeat Sessions. Am J Neuroradiol (2015) 36(2):384–90. 10.3174/ajnr.A4114 PMC7965670 25258365

